# Statistical evaluation of main extraction parameters in twenty plant extracts for obtaining their optimum total phenolic content and its relation to antioxidant and antibacterial activities

**DOI:** 10.1002/fsn3.2288

**Published:** 2021-05-06

**Authors:** Dler H. Kadir

**Affiliations:** ^1^ Department of Statistics College of Administration and Economics Salahaddin University‐Erbil Kurdistan Region Iraq

**Keywords:** ANOVA test and box plot, Biological activities, Extraction parameters, Total phenolic content

## Abstract

The main extraction parameters were statistically evaluated for 20 medicinal plants to obtain the optimum conditions for maximum extraction of total phenolic content (TPC) in each plant. Among various extraction parameters, pH, temperature, and concentration at different levels were studied. The results were analyzed using the analysis of variance to achieve the optimum conditions of phenolic extraction for all plants. Also, investigation of the optimum antioxidant (AnOX) activities using DPPH (2,2‐diphenyl‐1‐picrylhydrazyl) method and antibacterial potential against common pathogenic bacteria of *Staphylococcus* *aureus, Escherichia coli, Pseudomonas spp*., and *Candida spp*. through disk diffusion method for the extract of all plants under the optimum total phenolic concentration of each plant extract confirmed a direct relation among bioactivity and TPC.

## Graphical abstract

The main core of the MS is about the Statistical evaluation of main extraction parameters in twenty plant extracts for obtaining their optimum total phenolic content and its relation to antioxidant and antibacterial activities.

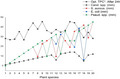

## 1 INTRODUCTION

During the ancient times, plant resources are involving an integral part of human society as which after fulfilling the primary needs, man has sought for a suitable remedy among plants for curing various diseases. Besides always role of medicinal plants at forefront virtually all cultures of civilizations, they as rich resources of traditional medicines have been playing an essential role in the development of human culture. For the large population of the world, nowadays medicinal plants present a dominant role in the healthcare system and financial aids. In diversified industries and development of new drug discovery, the contribution of plants and especially medicinal plants is remarkable as their roles have proved in coping with a number of deadly diseases such as cancer, hepatitis, and AIDS etc, Hamburger and Hostettmann, 1991; Harrison, 1998; Ahmad Dar et al., 2017; Keskin, 2018; Dehdari and Hajimehdipoor, 2018. Following the latest scientific estimations, a very large spectrum of plant species including new ones is characterized each year. According to a report released by the World Health Organization (WHO), among of these numbers of the plants, those can be used for treatment is around 20,000. A statistic obtained based on the reports revealed that plant products used by patients of chronic medical conditions including cancers (2%), liver diseases (21%), HIV (22%), asthma (24%), and rheumatologic disorders (26%), Tolossa and Megersa, 2018; Varga et al., 2018; Anand et al., 2019. The historical development of the health and disease has led to the alternative medicines and its practices to be conveyed to today's societies. Therefore, phytotherapy as one of the alternative medicine practices has become basic subjects of many scientific studies about treatment with plants, Spiridon et al., 2011; Emad, 2013; Efferth and Kaina, 2011.

The bioactive phytochemicals show significant applications in medicine in which their curing effects are used throughout the world. Among the medicinal secondary metabolites inside the plants, phenolic compounds have several biological effects, such as anti‐inflammatory, antibacterial, and antioxidant properties and can play an important role in the prevention of many diseases. Antioxidant phenolic compounds exhibit a wide range of bioactivities such as reducing LDL, inhibiting platelet aggregation, removal of free radicals, and preventing cell proliferation, Lin Song et al. 2010; Ghasemi et al. 2013; Clark, 1996; Fridlender et al., 2015. Typical antioxidant phenolics are predominantly phenolic acids and flavonoids Kaur and Mondal, 2014; Harkey et al., 2001; Jones, 1998. Phenolic acids, including caffeic acid, ferulic acid, and vanillic acid, are widely distributed have been repeatedly implicated as natural antioxidants. The redox properties of phenolics enable them to act as reducing agents, hydrogen donors, singlet oxygen quenchers, and metal chelating potential. In the other word, phenolic compounds are good electron donors because their hydroxyl groups can directly contribute to antioxidant action, Kaur and Mondal, 2014; Lateef Molan et al. 2012; Li et al., 2018; Sembiring et al., 2018. The current study focused on the statistically investigation of the optimal extraction conditions of polyphenolic compounds in some of the most popular medicinal plants and highlighting the effect of these optimal parameters on obtaining the maximum phenolic extraction and maximum antioxidant activity of their extracts during a time span ranging 24h to 1 month. Further, following the obtaining the optimum phenolic content for each plant, their potential for antibacterial activities was studied against common pathogenic bacteria of *Staphylococcus* *aureus, Escherichia coli, Pseudomonas spp*., and *Candida spp*.

## 2 EXPERIMENTAL

### 2.1 Materials and instrumentations

All chemicals used in current study were purchased from Merck and Aldrich companies. UV‐visible spectral analysis was recorded on a double‐beam spectrophotometer (Super Aquarius) to monitor the TPC and An. Ox activity.

### 2.2 Total phenolic content (TPC)

The *Folin–Ciocalteu* method was employed by Alhakmani, and his collaborators with some modifications were used to measure the TPC of each medicinal plant at studied conditions, Alhakmani et al. 2013. Generally, 1.0 ml of plant aqueous extract in 60 ml distilled water was mixed to 5.0 ml of *Folin–Ciocalteu* reagent. After some minutes, 15.0 ml Na_2_CO_3_ (20%) was added to the mixture. Finally, the absorbance of the mixture was measured at 760 nm after 2h and the TPC was reported as mg of gallic acid equivalent (GAE) and the influence of each extraction parameters at a special range such as various pHs, temperatures, and concentrations were separately recorded on the rate of phenolic content of each plant, Table 1. The optimum conditions for achieving the maximum phenolic content of plant extracts then were evaluated using the statistical calculations.

**TABLE 1:** Main parameters influenced on the TPC for twenty medicinal plants at various experimental situations

No.	Plant species	Family	TPC* at 25°C	TPC at 50°C	TPC at 75°C	TPC at 100°C	TPC at 20% Mix.*	TPC at 10% Mix.	TPC at 5% Mix.	TPC at pH 3	TPC at pH 5	TPC at pH 7	TPC at pH 9
1	*Adinandra nitida*	*Theaceae*	10.5	13.2	18.4	8.3	19.3	13.6	9.1	6.2	17.3	15.8	14.1
2	*Adinandra dumosa*	*Theaceae*	6.5	11.3	9.5	9.1	6.8	9.4	7.2	4.8	4.8	5.1	6.2
3	*Ageratum conyzoides L*	*Asteraceae*	7.3	6.4	8.5	9.6	11.5	11.1	10.4	6.1	7.6	9.1	8.3
4	*Adenanthera pavonina L*	*Leguminosae*	5.7	8.9	9.1	8.6	9.4	8.2	6.0	4.7	6.1	8.6	7.7
5	*Aleurites moluccana*	*Euphorbiaceae*	6.5	6.8	7.9	8.8	8.1	7.5	6.9	6.3	6.4	6.6	6.5
6	*Alocasia macrorrhiza*	*Araceace*	7.3	7.9	8.9	8.7	8.8	7.9	6.1	5.2	6.1	6.6	7.6
7	*Euphorbia condylocarpa*	*Euphorbiaceae*	10.3	11.7	14.9	13.8	17.1	18.2	16.3	10.0	10.8	16.6	16.1
8	*Artocarpus altilis*	*Moraceae*	7.7	8.7	6.8	5.3	5.3	9.2	6.3	3.1	5.4	6.3	10.1
9	*Barringtonia asiatica*	*Barringtoniaceae*	5.3	5.6	5.6	4.3	6.5	6.8	5.8	3.1	5.1	6.5	6.5
10	*Barringtonia racemosa*	*Lecythidaceae*	6.1	7.3	8.9	8.6	7.2	7.0	6.1	5.1	5.6	7.7	8.7
11	*Bruguiera gymnorrhiza*	*Rhizophoraceae*	3.7	5.3	5.3	4.6	6.7	6.1	5.1	5.2	5.9	6.8	6.1
12	*Capsicum frutescens*	*Solanaceae*	7.1	8.9	11.9	10.7	11.1	12.6	10.8	6.3	8.9	9.9	9.1
13	*Cassytha filiformis L*.	*Cassythaceae*	12.1	14.2	15.6	13.1	17.2	16.5	15.2	7.3	10.6	13.2	13.3
14	*Centella asiatica L*.	*Apiaceae*	6.2	8.5	8.5	6.3	10.1	9.4	6.1	6.7	7.2	8.4	8.2
15	*Commelina diffusa*	*Commelinaceae*	5.1	6.5	7.6	7.6	8.8	7.9	7.6	6.5	7.6	8.8	8.2
16	*Commersonia bartramia*	*Commersonia*	4.3	6.5	8.8	6.5	11.4	10.2	8.6	6.8	7.2	7.7	7.3
17	*Cordia subcordata*	*Boraginaceae*	6.4	9.1	14.6	11.2	16.1	13.2	10.1	5.1	9.8	13.4	11.6
18	*Cordyline fruticosa*	*Asparagaceae*	9.6	12.3	20.3	16.1	24.2	21.3	15.2	11.5	14.3	16.1	13.9
19	*Euodia hortensis*	*Rutaceae*	4.1	5.3	4.6	3.3	8.3	7.6	6.4	4.6	5.2	5.8	4.8
20	*Euphorbia fidjiana Boiss*	*Euphorbiaceae*	8.6	10.2	14.6	12.1	14.8	13.8	13.8	8.4	10.3	9.6	9.0

Abbreviation: TPC*: Total phenolic content in GAE (mg/g dry weight); %Mix*.:(W/V) % Mixture where W is the weight of the dried plant in g, and V is the volume of solvent in mL.

### 2.3 Calculation of the optimal antioxidant activity

After obtaining the optimal conditions to extract the maximum TPC for each plant, the antioxidant activity of each medicinal plant was monitored against 2,2‐diphenyl‐1‐picrylhydrazyl (DPPH). Briefly, a 1:1 ratio of each plant optimum aqueous extract at different concentrations were mixed with freshly prepared DPPH solution, and after their keeping in the dark for 30 min, the absorbance at 517 nm was recorded using UV‐vis spectrophotometer in contrast with the gallic acid as control. The optimal antioxidant activity for each plant extract using DPPH method was calculated as below:

$$\text{Radical Scavenging Activity}\;\left(\text{RSA}\%\right)\;=\;\left[\left(A_{c}‐{\;A}_{t}\right)\;/{\;A}_{c}\right]\;\times 100.$$

where A_c_ and A_t_ are the absorbance of control and sample, respectively. The results of DPPH method for each plant extract at optimal conditions to decrease the absorbance of DPPH radicals were reported as scavenging activity (%). The assay to monitor the antioxidant activity of each plant extract in optimal conditions was repeated during a time span ranging 24h to 1 month, Table S2.

### 2.4 Investigation of the antibacterial activity

Following the obtain of total phenolic content (TPC) for each of the studied plant shown in Table 1, the antibacterial activity for each plant was monitored at mentioned optimal conditions. Briefly, the disk diffusion method was employed in which 100 µl of each plant aqueous extract and Chloramphenicol at the same condition were loaded on paper disks (6 mm D) and dried; then, they were placed on the Muller Hinton Agar plates including bacterial cultures (100 µl). Each plate was inoculated at 37°C for 24 hr after incubation. Finally, the diameter of inhibition zones was measured and tabulated to find the MIC.

### 2.5 Statistical analysis

The test of examining normality was detected to conduct the comparison test. The data showed that the assumption of normality was met and ANOVA test was applied to find the difference between the groups at both aspects, and LSD post hoc test was implemented for the pairs. Plus, *t* test was used for comparing two groups. The statistical software SPSS v25 was used to generate all the results and outputs for this study with 0.05 significant value.

## 3 RESULTS AND DISCUSSION

### 3.1 Statistical analysis to monitor the optimal extraction parameters

To reach the objective of the study and explore, we have carried out several statistical techniques for the accuracy of the methods and the comparison between the groups of implanting. An intensive descriptive statistic including tables and graphs were generated to show the nature of the data and detect patterns among the dataset, Table 1.

**FIGURE 1:**
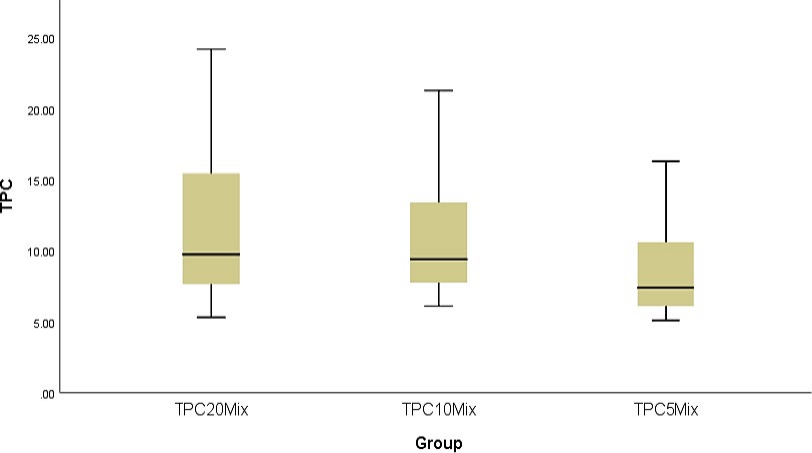
Box plot chart for concentration for each level

Table S2 shows that the highest TPC recorded in the study was at 20% concentration with 11.435, followed by 10% with 10.875 and the lowest mean values were for at 5% with 8.955. This means that the more degree of concentration leads to the more increased TPC. To see if there is any statistically significant among the degree of concentration percentage used in this study, ANOVA test was carried out since there are more than two groups to compare their mean values and Figure 1 proves that the data are normally distributed. Table S3 states that there is indeed not statistical difference between the three degrees as the p‐value of ANOVA test is greater than 0.05.

**FIGURE 2:**
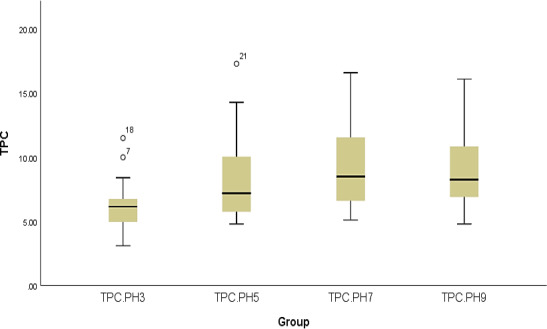
Box plot chart for pH data for each degree

Similar to previous technique, we are seeking to identify which degree of pH recorded the highest TPC. Mean value can lead us to find, and at pH7, the largest mean value was found with 9.43, and a very close to this was at pH 9 with 9.16, whereas the smallest value was at pH3 with 6.15 only. Again, we use ANOVA due to meeting our requirements, the very interested one is normality distribution as shown in Figure 2 assuming normality. Table S4 states that there is indeed statistical difference between the three degrees as the p‐value of ANOVA test is less than 0.05.

Since the p‐value of ANOVA test in Table S5 was less than 0.05, we can report that there is statistically significant between the different types of pH degree provided to the plants in the study. Like before, LSD test was computed for each pair to find out the exact difference. Table S6 illustrates that they were all found to be statistically significant regarding to their mean values except between pH 7 and pH 9 as we already discussed in the descriptive phase where their mean values were quite close to each other.

**FIGURE 3:**
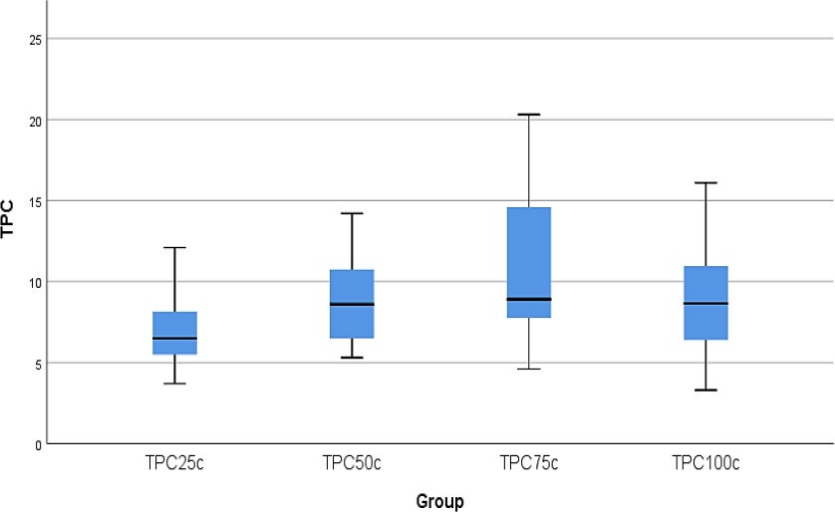
Box plot of TPCs at different Temperature

Table S7 provides us information about how TPC changed according to varying degree of temperature. four degrees tried and the value of TPCs at each degree was recorded accordingly. At the beginning, it was thought along with rising temperature, TPC increased, and however, it was not true since it peaked at 75 C with mean value 10.52 and then fall to 8.83 at 100 C. Figure 3 forces us to go ahead with using ANOVA test to compare the mean values of TPC between each degree of temperature. Although there seemed to be highly statistically significant among the mean values, yet this cannot be confirmed without further tests such as ANOVA.

The result of ANOVA test stated in Table S8 tells us that there is of course difference among the means due to having p‐value less than 0.05. LSD was again used to identify the difference between the pairs of degrees, Table S9. Statistically significant difference among degrees occurred between all of them expect 25 C and 75 C since their p‐values were less than 0.05.

### 3.2 Investigation of the optimal TPC and AnOX activity

Through this study, some main extraction parameters were assessed to extract of phenolic compounds of twenty widely distributed medicinal plants and the results were defined as total phenolic contents of the plant extracts, Figure S1. Also, as the antioxidant activity shows a very closed relation to the TPC of the plants, it can be defined as TPC dependent factor. Thus, along with evaluation of the TPC for the plants at optimum conditions, the optimum AnOX activity was also reported. The discussed parameters during this study influenced on the TPC and AnOX activity were pH, temperature, and concentration (plant ration (g)/ 100 ml DW) where all of them were considered during a time span from 24h to 1 month to study of the repeatability and accuracy of our results, Table S8. As the statistically evaluation of the results showed in previous sections, the optimum extraction parameters were pH 7, temperature 75°C, and concentration 20%.

According to Table 1, for most of the plants there is a decreasing rate for TPC of the plants for acidic or basic pH, temperatures less or more than 75°C, and also concentrations less than 20%. Of course, this results are probably due to the decomposition of phenolic compounds of the plants in acidic or basic media and also occurring deformation and decomposition processes for temperatures more than 75°C. Besides the TPC in temperatures more than optimal condition, for temperatures less than 75°C, the kinetic energy of the solvent molecules penetrating to the plant tissues or plant cells to movement of phytochemicals from plant cytoplasm to the solvent medium is slow; thus, the rate of phenolic compounds for temperatures less than optimum temperature is decreased. In case of concentration as another important factor on TPC and AnOX activity, the experimental results show that increasing the ratio of plant powder inside 100 ml distilled water (DW) caused to increasing the rate of TPC extraction which the process is for availability of more plant phytochemicals and migration them from plant cell cytoplasm (high concentration medium) to solvent as low concentration media due to the osmosis process. Following the obtained results from investigation of TPC and AnOX activity at optimum conditions, generally, with increasing the TPC amount, the rate of AnOX activity is ascended. Also, passing the time caused to decreasing the rate of phenolic content and AnOX activity in studied plants which is maybe for appearance of some deformation processes during the time. Although in most of the plants this reality can be observed but, in some plants, it is a reverse phenomenon such as Table 10, entries 3, 11, and 14 where the AnOX activity is almost constant with passing the time that it is probably for presence of more stable phytochemicals inside the plant. For entry 13 in Table 10, there is a strong descending slope for AnOX activity with passing the time as which after 1 month there was no AnOX activity can be detected by our method.

**TABLE 10:** TPC and AnOX activity reported at optimal conditions*

Entry	Plant species	Family	Opt. TPC*. After 24h	Opt. TPC. After 7 days	Opt. TPC. After 1 month	Opt. AnOX*. after 24 hr	Opt. AnOX. after 7 days	Opt. AnOX. after 1 month
1	*Adinandra nitida*	*Theaceae*	28.1	25.1	19.1	57.6	53.4	22.1
2	*Adinandra dumosa*	*Theaceae*	26.9	23.5	14.7	53.4	49.1	31.1
3	*Ageratum conyzoides L*	*Asteraceae*	27.4	25.2	6.7	42.1	44.2	40.3
4	*Adenanthera pavonina L*	*Leguminosae*	35.2	29.2	8.3	22.8	14.3	13.9
5	*Aleurites moluccana*	*Euphorbiaceae*	26.8	26.6	17.1	67.6	56.7	37.6
6	*Alocasia macrorrhiza*	*Araceace*	37.2	33.8	22.1	37.9	33.7	22.5
7	*Euphorbia condylocarpa*	*Euphorbiaceae*	44.1	31.2	24.5	61.7	44.2	29.1
8	*Artocarpus altilis*	*Moraceae*	37.9	35.6	20.3	29.2	22.6	17.3
9	*Barringtonia asiatica*	*Barringtoniaceae*	45.5	41.5	15.6	74.6	71.3	36.4
10	*Barringtonia racemosa*	*Lecythidaceae*	36.3	34.9	19.7	92.7	88.6	56.3
11	*Bruguiera gymnorrhiza*	*Rhizophoraceae*	33.7	25.3	17.4	28.6	28.0	27.4
12	*Capsicum frutescens*	*Solanaceae*	37.3	29.1	26.3	44.1	22.1	16.1
13	*Cassytha filiformis L*.	*Cassythaceae*	32.4	34.7	25.1	32.6	11.9	NR
14	*Centella asiatica L*.	*Apiaceae*	36.5	31.1	18.6	29.3	29.5	28.9
15	*Commelina diffusa*	*Commelinaceae*	45.4	36.6	19.8	26.4	22.4	17.3
16	*Commersonia bartramia*	*Commersonia*	44.4	16.3	21.4	49.3	16.5	11.2
17	*Cordia subcordata*	*Boraginaceae*	36.8	29.4	24.9	82.6	44.8	29.3
18	*Cordyline fruticosa*	*Asparagaceae*	29.1	22.2	20.7	67.4	59.3	39.5
19	*Euodia hortensis*	*Rutaceae*	34.4	26.9	25.1	56.3	33.1	26.4
20	*Euphorbia fidjiana Boiss*	*Euphorbiaceae*	28.2	20.9	11.3	27.3	19.5	14.2

Abbreviation: Opt. TPC*: Total Phenolic Content at optimum conditions; Opt. AnOX; Antioxidant activity at optimum conditions according scavenging activity (%); Optimal conditions are pH 7, temperature 75°C and concentration 20%; Opt. AnOX: the measurement was presented based on RSA%.

### 3.3 Investigation of the antimicrobial activity

**FIGURE 4:**
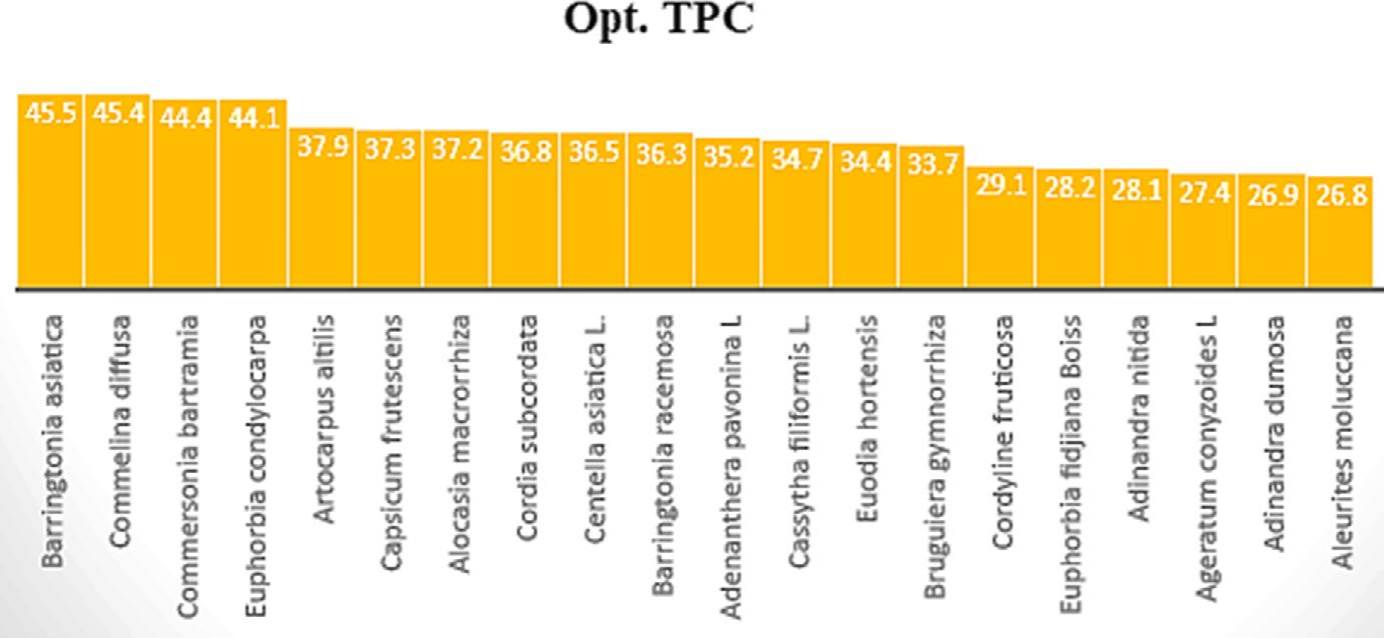
The Opt.TPC in GAE (mg/g dry weight) of the plant extracts obtained at optimal extraction condition of pH, temperature (°C) and Concentration (*W*/*V*)% where *W* is the weight of the dried plant in g and *V* is the volume of solvent in mL

**FIGURE 5:**
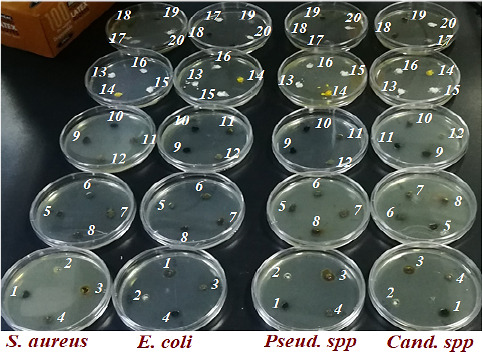
Antibacterial potential of all plant extracts including Opt. TPC against current pathogenic bacteria

As it was mentioned for investigation of the AnOX activities for studied plants after evaluation of the extraction parameters to extract of phenolic compounds of twenty medicinal plant extracts, in this part, we try to evaluate the antibacterial activities of the mentioned plant extracts including optimal amount of total phenolic content (TPC), Figure 4. As it can be seen in Table 11, we select the plant extract at their maximum phenolic contents which this maximum was obtained for all of them after 24 hr except *Cassytha filiformis L*. (entry 13) which its phenolic content was reached to a maximum amount after one week. Based on our experimental observation, the antimicrobial activity of the plants shows a direct relation to their phenolic content in which as the phenolic content is increased the antimicrobial activity is also improved, Figure 5. The result is probably referred to the considerable antimicrobial effects of phenolic compounds against a large spectrum of pathogenic bacteria comparing other bioactive phytochemicals.

**TABLE 11:** Antimicrobial activities of plant extracts including Opt. TPC*

Entry	Plant species	Family	Opt. TPC*. After 24h	S. aureus (mm)*	*E. coli (mm)*	*Pseud. spp*. *(mm)*	*Cand*. *spp*. *(mm)*
1	*Adinandra nitida*	*Theaceae*	28.1	18 mm	21 mm	18 mm	24 mm
2	*Adinandra dumosa*	*Theaceae*	26.9	17 mm	20 mm	17 mm	21 mm
3	*Ageratum conyzoides L*	*Asteraceae*	27.4	23 mm	23 mm	27 mm	23 mm
4	*Adenanthera pavonina L*	*Leguminosae*	35.2	31 mm	31 mm	31 mm	31 mm
5	*Aleurites moluccana*	*Euphorbiaceae*	26.8	24 mm	19 mm	24 mm	22 mm
6	*Alocasia macrorrhiza*	*Araceace*	37.2	33 mm	35 mm	33 mm	32 mm
7	*Euphorbia condylocarpa*	*Euphorbiaceae*	44.1	37 mm	40 mm	40 mm	38 mm
8	*Artocarpus altilis*	*Moraceae*	37.9	31 mm	32 mm	34 mm	35 mm
9	*Barringtonia asiatica*	*Barringtoniaceae*	45.5	38 mm	38 mm	41 mm	42 mm
10	*Barringtonia racemosa*	*Lecythidaceae*	36.3	28 mm	31 mm	31 mm	30 mm
11	*Bruguiera gymnorrhiza*	*Rhizophoraceae*	33.7	26 mm	27 mm	26 mm	26 mm
12	*Capsicum frutescens*	*Solanaceae*	37.3	31 mm	32 mm	30 mm	32 mm
13	*Cassytha filiformis L*.	*Cassythaceae*	34.7 (after 7 days)	25 mm	24 mm	24 mm	27 mm
14	*Centella asiatica L*.	*Apiaceae*	36.5	28 mm	28 mm	32 mm	29 mm
15	*Commelina diffusa*	*Commelinaceae*	45.4	37 mm	38 mm	41 mm	41 mm
16	*Commersonia bartramia*	*Commersonia*	44.4	39 mm	40 mm	41 mm	41 mm
17	*Cordia subcordata*	*Boraginaceae*	36.8	29 mm	29 mm	32 mm	30 mm
18	*Cordyline fruticosa*	*Asparagaceae*	29.1	21 mm	23 mm	23 mm	22 mm
19	*Euodia hortensis*	*Rutaceae*	34.4	26 mm	26 mm	28 mm	26 mm
20	*Euphorbia fidjiana Boiss*	*Euphorbiaceae*	28.2	20 mm	22 mm	22 mm	27 mm

*Inhibition diameter

Abbreviation: Opt. TPC*: optimal total phenolic content

## 4 CONCLUSIONS

This study shows the relation of the main extraction parameters of phenolic compounds such as pH, temperature, and concentration to obtain maximum total phenolic content in twenty plant extracts. The methods used in this study contained both experimental methods in different conditions of the mentioned parameters and theoretical study using the assessment of examining normality using ANOVA and LSD post hoc tests. The results showed that the best condition to achieve the maximum TPC for plants were pH 7, temperature 75°C, and concentration 20% where employing these optimal conditions caused to minimum deformation and decomposition side processes. Furthermore, the antioxidant and antibacterial activities of the plant extracts including the optimal amounts of phenolics were investigated to demonstrate the effect of phenolic content on their biological activity in which the results showed that increasing the phenolic content in plant extracts caused to enhance the antioxidant and antibacterial activities.

## CONFLICT OF INTEREST

The authors declare that they do not have any conflict of interest.

## ETHICAL APPROVAL

This study does not involve any human or animal testing.

5

**FIGURE 6:**
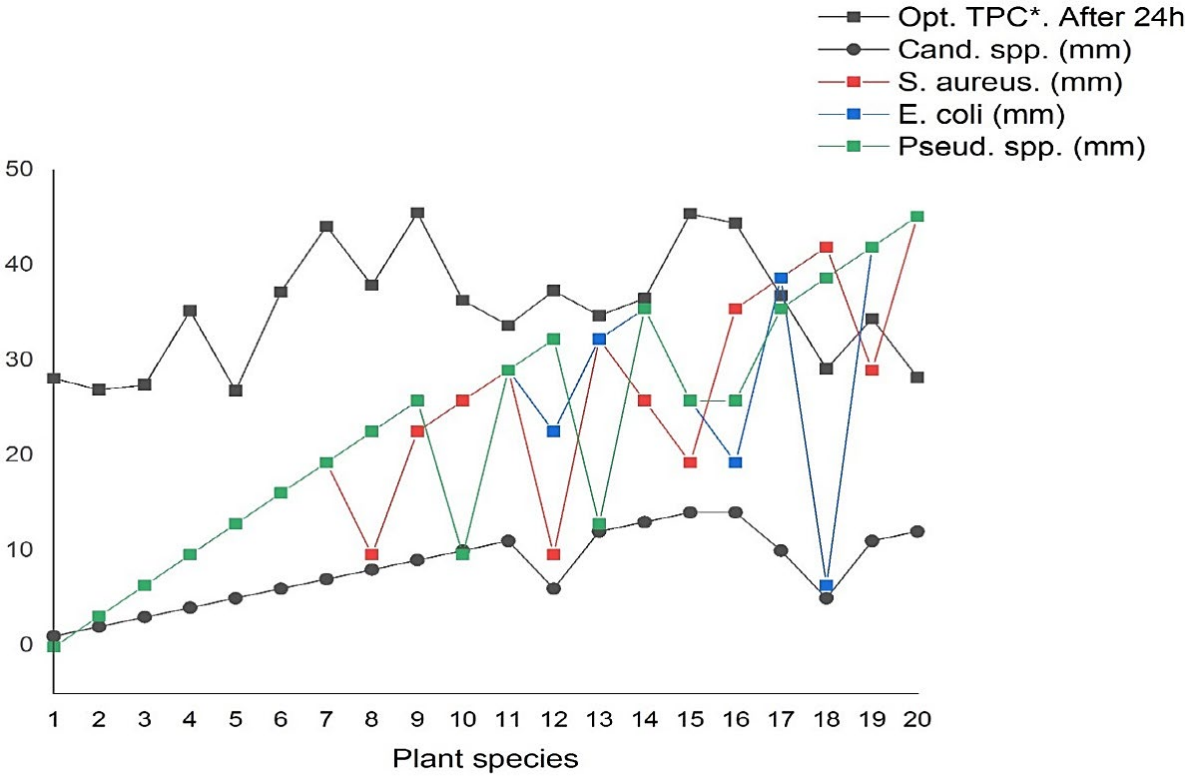
Relation of antimicrobial activities of plant extracts and their optimal total phenolic content

## Supporting information

Supplementary Material
