# Supplementary material for: Statistical evaluation of main extraction parameters in twenty plant extracts for obtaining their optimum total phenolic content and its relation to antioxidant and antibacterial activities
Source: Food Sci Nutr. 2021 May 6;9(7):3491–9. doi: 10.1002/fsn3.2288 (PMC8269642; doi:10.1002/fsn3.2288)
Supplement: Supplementary file 1 — Supplementary Material [file FSN3-9-3491-s001.docx]

**Supporting Information**

**Statistical evaluation of main extraction parameters in twenty plant extracts for obtaining their optimum total phenolic content and its relation to antioxidant and antibacterial activities**

Dler H. Kadir^*^

*Department of Statistics, College of Administration and Economics, Salahaddin University-Erbil, Kurdistan Region, Iraq*

**Corresponding author email:* [*dler.kadir@su.edu.krd*](mailto:dler.kadir@su.edu.krd)*,* [*DHKadir2@gmail.com*](mailto:DHKadir2@gmail.com)

**Table 2.** Descriptive statistics for concentration data

|  | N | Mean | Std. Deviation | Std. Error | 95% Confidence Interval for Mean | |
| --- | --- | --- | --- | --- | --- | --- |
|  |  |  |  |  | Lower Bound | Upper Bound |
| TPC at 20% Mix*. | 20 | 11.4350 | 5.06757 | 1.13314 | 9.0633 | 13.8067 |
| TPC at 10% Mix. | 20 | 10.8750 | 4.15108 | .92821 | 8.9322 | 12.8178 |
| TPC at 5% Mix. | 20 | 8.9550 | 3.57690 | .79982 | 7.2810 | 10.6290 |
| Total | 60 | 10.4217 | 4.36877 | .56401 | 9.2931 | 11.5502 |

**Table 3.** ANOVA test for Mix data

|  | Sum of Squares | df | Mean Square | F | Sig. |
| --- | --- | --- | --- | --- | --- |
| Between Groups | 67.669 | 2 | 33.835 | 1.822 | .171 |
| Within Groups | 1058.413 | 57 | 18.569 |  |  |
| Total | 1126.082 | 59 |  |  |  |
|  | | | | | |

**Table 4.** Descriptive Statistics for pH data

|  | N | Mean | Std. Deviation | Std. Error | 95% Confidence Interval for Mean | |
| --- | --- | --- | --- | --- | --- | --- |
|  |  |  |  |  | Lower Bound | Upper Bound |
| TPC.PH3 | 20 | 6.1500 | 2.04772 | .45788 | 5.1916 | 7.1084 |
| TPC.PH5 | 20 | 8.1100 | 3.28215 | .73391 | 6.5739 | 9.6461 |
| TPC.PH7 | 20 | 9.4300 | 3.61417 | .80815 | 7.7385 | 11.1215 |
| TPC.PH9 | 20 | 9.1650 | 3.08771 | .69043 | 7.7199 | 10.6101 |
| Total | 80 | 8.2138 | 3.27390 | .36603 | 7.4852 | 8.9423 |

**Table 5.** the ANOVA test for pH data

|  | Sum of Squares | df | Mean Square | F | Sig. |
| --- | --- | --- | --- | --- | --- |
| Between Groups | 133.079 | 3 | 44.360 | 4.724 | .004 |
| Within Groups | 713.675 | 76 | 9.390 |  |  |
| Total | 846.755 | 79 |  |  |  |

**Table 6.** Multiple Comparison test for pairs

| (I) Group | (J) Group | Mean Difference (I-J) | Std. Error | Sig. | 95% Confidence Interval | |
| --- | --- | --- | --- | --- | --- | --- |
|  |  |  |  |  | Lower Bound | Upper Bound |
| TPC.PH3 | TPC.PH5 | -1.96000* | .96904 | .047 | -3.8900 | -.0300 |
|  | TPC.PH7 | -3.28000* | .96904 | .001 | -5.2100 | -1.3500 |
|  | TPC.PH9 | -3.01500* | .96904 | .003 | -4.9450 | -1.0850 |
| TPC.PH5 | TPC.PH3 | 1.96000* | .96904 | .047 | .0300 | 3.8900 |
|  | TPC.PH7 | -1.32000 | .96904 | .177 | -3.2500 | .6100 |
|  | TPC.PH9 | -1.05500 | .96904 | .280 | -2.9850 | .8750 |
| TPC.PH7 | TPC.PH3 | 3.28000* | .96904 | .001 | 1.3500 | 5.2100 |
|  | TPC.PH5 | 1.32000 | .96904 | .177 | -.6100 | 3.2500 |
|  | TPC.PH9 | .26500 | .96904 | .785 | -1.6650 | 2.1950 |
| TPC.PH9 | TPC.PH3 | 3.01500* | .96904 | .003 | 1.0850 | 4.9450 |
|  | TPC.PH5 | 1.05500 | .96904 | .280 | -.8750 | 2.9850 |
|  | TPC.PH7 | -.26500 | .96904 | .785 | -2.1950 | 1.6650 |

**Table 7.** Descriptive Statistics for temperature data

|  | N | Mean | Std. Deviation | Std. Error | 95% Confidence Interval for Mean | |
| --- | --- | --- | --- | --- | --- | --- |
|  |  |  |  |  | Lower Bound | Upper Bound |
| TPC* at 25°C | 20 | 7.02 | 2.251 | .503 | 5.97 | 8.07 |
| TPC at 50°C | 20 | 8.73 | 2.672 | .597 | 7.48 | 9.98 |
| TPC at 75°C | 20 | 10.52 | 4.431 | .991 | 8.44 | 12.59 |
| TPC at 100°C | 20 | 8.83 | 3.336 | .746 | 7.27 | 10.39 |
| Total | 80 | 8.77 | 3.447 | .385 | 8.01 | 9.54 |

**Table 8.** The ANOVA test for Temperature degree used for measuring TPC

|  | Sum of Squares | df | Mean Square | F | Sig. |
| --- | --- | --- | --- | --- | --- |
| Between Groups | 122.253 | 3 | 40.751 | 3.793 | .014 |
| Within Groups | 816.421 | 76 | 10.742 |  |  |
| Total | 938.675 | 79 |  |  |  |

**Table 9.** Multiple Comparison test for each pair

| (I) Group | (J) Group | Mean Difference (I-J) | Std. Error | Sig. | 95% Confidence Interval | |
| --- | --- | --- | --- | --- | --- | --- |
|  |  |  |  |  | Lower Bound | Upper Bound |
| TPC25c | TPC50c | -1.710 | 1.036 | .103 | -3.77 | .35 |
|  | TPC75c | -3.495* | 1.036 | .001 | -5.56 | -1.43 |
|  | TPC100c | -1.810 | 1.036 | .085 | -3.87 | .25 |
| TPC50c | TPC25c | 1.710 | 1.036 | .103 | -.35 | 3.77 |
|  | TPC75c | -1.785 | 1.036 | .089 | -3.85 | .28 |
|  | TPC100c | -.100 | 1.036 | .923 | -2.16 | 1.96 |
| TPC75c | TPC25c | 3.495* | 1.036 | .001 | 1.43 | 5.56 |
|  | TPC50c | 1.785 | 1.036 | .089 | -.28 | 3.85 |
|  | TPC100c | 1.685 | 1.036 | .108 | -.38 | 3.75 |
| TPC100c | TPC25c | 1.810 | 1.036 | .085 | -.25 | 3.87 |
|  | TPC50c | .100 | 1.036 | .923 | -1.96 | 2.16 |
|  | TPC75c | -1.685 | 1.036 | .108 | -3.75 | .38 |
